# Hydrocarbon degradation abilities of psychrotolerant *Bacillus* strains

**DOI:** 10.3934/microbiol.2017.3.467

**Published:** 2017-06-13

**Authors:** Fulya Kolsal, Zeynep Akbal, Fakhra Liaqat, Oğuzhan Gök, Delia Teresa Sponza, Rengin Eltem

**Affiliations:** 1 Department of Bioengineering, Faculty of Engineering, Ege University, 35100, İzmir, Turkey; 2 Department of Biotechnology, Graduate School of Natural and Applied Sciences, Ege University, 35100, İzmir, Turkey; 3 Department of Environmental Engineering, Engineering Faculty, Aksaray University, Aksaray, Turkey; 4 Department of Environmental Engineering, Engineering Faculty, Dokuz Eylül University, Buca, Kaynaklar Campus, 35160, İzmir, Turkey

**Keywords:** hydrocarbons, PAHs, psychrotolerant, *Bacillus*, GC-MS

## Abstract

Biodegradation requires identification of hydrocarbon degrading microbes and the investigation of psychrotolerant hydrocarbon degrading microbes is essential for successful biodegradation in cold seawater. In the present study, a total of 597 *Bacillus* isolates were screened to select psychrotolerant strains and 134 isolates were established as psychrotolerant on the basis of their ability to grow at 7 °C. Hydrocarbon degradation capacities of these 134 psychrotolerant isolate were initially investigated on agar medium containing different hydrocarbons (naphthalene, n-hexadecane, mineral oil) and 47 positive isolates were grown in broth medium containing hydrocarbons at 20 °C under static culture. Bacterial growth was estimated in terms of viable cell count (cfu ml^−1^). Isolates showing the best growth in static culture were further grown in presence of crude oil under shaking culture and viable cell count was observed between 8.3 × 10^5^–7.4 × 10^8^ cfu ml^−1^. In the final step, polycyclic aromatic hydrocarbon (PAH) (chrysene and naphthalene) degradation yield of two most potent isolates was determined by GC-MS along with the measurement of pH, biomass and emulsification activities. Results showed that isolates Ege B.6.2i and Ege B.1.4Ka have shown 60% and 36% chrysene degradation yield, respectively, while 33% and 55% naphthalene degradation yield, respectively, with emulsification activities ranges between 33–50%. These isolates can be used to remove hydrocarbon contamination from different environments, particularly in cold regions.

## 1. Introduction

Use of hydrocarbons by microorganisms for their growth is called biodegradation of hydrocarbons and the first report about hydrocarbon degradation ability of microorganisms was published in 1895 by Miyoshi. After that hydrocarbon degradation by different species of bacteria, fungus and yeasts have been reported and bacteria were found to be the best group of microorganisms for hydrocarbon biodegradation [1]. Compared to the other physical and chemical methods including photolysis, volatilization, photo-oxidation, chemical oxidation, adsorption combustion, landfill and ultrasonic decomposition, biodegradation is anticipated to be a cost-effective and environmentally friendly alternative for removal of hydrocarbons [2].

Hydrocarbons contain only carbon and hydrogen atoms in their structure and many of these are industrially important compounds. Crude oil as a hydrocarbon mixture is the most imperative hydrocarbon. Benzene, naphthalene, acetylene and many other commonly used chemicals contain hydrocarbon structures [3],[4]. Polycyclic aromatic hydrocarbons (PAHs) are common pollutants in urban atmospheres [5],[6]. The low-aqueous solubility of hydrocarbons including aliphatics, mono and polycyclic-aromatics, increases their recalcitrance to bacteria and limits their biodegradation which leads to continuous contamination of several soil areas and sea water along with harmful effects on the fauna and flora [7]. The presence of hydrocarbon degrading bacteria in the crude oil-contaminated seawater is very important to establish an effective method to bioremediate the contaminated marine environments. PAHs [8]. It was observed that low temperature evidently inhibits the diesel oil-degrading ability of bacteria, since the fluidity of cell membranes is remarkably decreased at low temperature and the exchange of intracellular and extracellular masses is restrained [8].

Microbial degradation is considered to be the key method involved in degradation of PAH [9]. Thus, more and more research interests are turning to the biodegradation of PAHs. Some microorganisms can utilize PAHs as a source of carbon and energy to degrade PAHs into carbon dioxide and water, or transform them to other nontoxic or less-toxic substances [10]. Microorganisms capable of living in high and low temperature or extreme environments are of great interest now a days. Investigation on biodegradation in cold seawater is becoming more and more essential, due to increased awareness of oil exploration in the arctic areas. However, the majority of research about biodegradation have been conducted at higher temperatures [11].

Hydrocarbon containing waste accumulation is a problem all over the world, particularly in cold or mild climate areas, as the biodegradation process of hydrocarbons is slower in low temperature. Considering these conditions psychrotolerant bacterial strains are becoming more important [12],[13]. The term psychrotolerant is actually used for the mesophilic microorganisms which are cold tolerant and can grow in psychrophilic temperature range. Optimum growth temperature for psychrophilic bacteria is 15 °C. While, psychrotolerant bacteria have a wider range as they can grow at 40 °C and can also survive below 10 °C [14],[15],[16]. The severe climate of northern areas retards the self-remediation of oil pollutants from the soils and water by the indigenous hydrocarbon-oxidizing microflora. Low average annual temperatures, insufficient aeration and shortage of biogenic elements are the key factors that limit the remediation process.

This study is designed to recover psychrotolerant isolates from “*Bacillus* Culture Collection of Bioengineering, Department”, Ege University, Izmir, Turkey, and to check the biodegradation abilities of these indigenous psychrotolerant isolates against different hydrocarbons. *Bacillus* species are easy to cultivate and effectual in utilizing complex carbon sources since they are capable of producing a variety of enzymes. Furthermore, this study is carried out to discover psychrotolerant biodegrading bacterial strains therefore, *Bacillus* species are preferred as they can grow under a wide range of temperatures. Although several works have been done on biodegradation of hydrocarbons and polycyclic aromatic compounds, but the majority of research has been conducted at high temperature and the use of psychrotolerant bacterial strains for this purpose is comparatively very less studied spot. In this work, we studied the ability of psychrotolerant microorganisms isolated from natural localities to degrade oil components in order to use them for bioremediation process in cold areas.

## 2. Materials and Methods

### 2.1. Screening of psychrotolerant *Bacillus* isolates

A total of 597 different *Bacillus* isolates from “*Bacillus* Culture Collection of Bioengineering, Department”, Ege University, Izmir, Turkey, were administered in this study. All these isolates were inoculated on CASO (Casein-peptone Soymeal-peptone Agar/Merck 105458) agar plates and incubated at 7 and 10 °C for 21 days to determine psychrotolerant isolates among these. Isolates showing growth at 7 °C were considered as psychrotolerant and further inoculated on CASO agar and incubated at 4 °C for 21 days to determine lower temperature limit and at 43 and 47 °C for 24–48 hours to determine the upper temperature limit [15].

### 2.2. Screening of hydrocarbon degradation abilities of psychrotolerant *Bacillus* isolates

To screen the naphthalene, hexadecane and mineral oil degradation capabilities of psychrotolerant *Bacillus* on solid medium, modified method of Demir et al. 2000 [17] was used. To prepare cell suspension for inoculum, bacterial cultures were inoculated in nutrient broth and incubated at 30 °C for 18 hours. At the end of incubation, the broth was centrifuged at 9000 rpm for 15 min and obtained pellet was resuspended in 10 ml of 0.85% sterile NaCl solution and centrifuged two times with this solution to wash the pellet. Washed pellet was resuspended in 10 ml sterile distilled water and used as inoculum. Solidified Bushnell Haas agar plates were poured with 200 µl sterile mineral oil, 200 µl sterile hexadecane and 200 µl of sterile naphthalene solution separately and poured solutions were evenly spread on the surface of the solidified agar with the help of sterile L-shaped rod. Prepared bacterial inoculums were inoculated on these plates and incubated at 20 °C for two weeks. Plates having bacterial growth after two weeks were considered positive for biodegradation of added hydrocarbons [17],[18].

### 2.3. Confirmation of hydrocarbon degradation abilities of psychrotolerant *Bacillus* isolates

Three sets of Bushnell Haas broth, two for each hydrocarbon was prepared in 150 ml flasks. The 1% hexadecane and 1% mineral oil were added before autoclaving the medium while naphthalene solution was separately autoclaved and aseptically added later. The 1% bacterial cell suspension or inoculum (prepared by the above mentioned method) was added and flasks were incubated statically at 20 °C for 21 days. The pH of medium was noted before inoculation and daily after incubation. At the end of the incubation viable count was measured by pour plate method [18],[19],[20].

### 2.4. Crude oil degradation testing

Isolates showing positive results for hydrocarbon degradation in broth medium under static culture conditions were selected to determine their abilities of degrading crude oil under shaking culture conditions. The Bushnell Haas broth was added with 1% crude oil and autoclaved to sterilize. Prepared medium was inoculated with bacterial cell suspensions of selected isolates and incubated at 20 °C at 200 rpm for 10 days. The pH of medium was noted before inoculation and daily after incubation. At the end of the incubation viable count was measured by pour plate method [21].

### 2.5. PAHs degradation testing and emulsifying activity

Two best hydrocarbon degrading *Bacillus* isolates were selected and further studied for their ability to degrade PAHs, chrysene and naphthalene were selected as PAH. Inoculum was prepared according to the above mentioned method and the Bushnell Haas broth was prepared by adding 50 mg L^−1^ chrysene and 100 mg L^−1^ naphthalene. The 1% bacterial suspension was inoculated into the broth and incubated at 20 °C and 200 rpm for 6 days. With two inoculated flasks containing broth added with chrysene (50 mg L^−1^) and naphthalene (100 mg L^−1^) two uninoculated flasks were also incubated to serve as controls. The sample was collected from the inoculated broth after every 24 hours to measure the pH, dry mass and viable count. To determine emulsifying activity, 2 ml sample was collected in two different tubes after 48 hours of incubation and 3 ml hexadecane was added in one tube while 3 ml mineral oil was added in the second tube. Tubes were vortexed for 2 min and incubated at room temperature for 24 hours. After incubation the height of the emulsion layer was measured and divided by total height and multiplied by 100 to calculate emulsion index (E24) [18],[22].

### 2.6. PAH analysis by GC-MS

GC-MS analysis was used to determine degradation yields of PAHs from the inoculated medium [2],[23]. Prior to GC analysis samples removed from inoculated and control broth were prepared by using Amberlite XAD-2 resin of 47 mm diameter. Samples were passed from the resin and obtained liquid was mixed with 30 ml acetone/hexane and kept at sonicator for 60 min. The supernatant of phase separation was collected in clean vial and reduced up to 2 ml with nitrogen gas [24]. For GC-MS analysis of PAHs, naphthalene (NAF) and chrysene (CHR), initial oven temperature was set to 50 °C for 1 minute and raised to 200 °C at the rate of 25 °C per minute. From 200 °C oven temperature is further raised to 300 °C at the rate of 8 °C per minute and 5.5 minutes retention time was selected. Ion source for Injector was determined and guadrapol temperatures were selected as 295, 300 and 180 °C. High purity Helium gas was used as carried gas at 1.5 ml^−1^, 45 cm s^−1^ flow rate [25].

## 3. Results and Discussion

Psychrophilic and psychrotolerant microorganisms are habitually found in low temperature ecosystems [26],[27]. Difficult elimination of hydrocarbon pollution in cold regions is a reason for growing research interests regarding psychrotolerant hydrocarbon degrading species [20],[28],[29]. Psychrotolerant *Bacillus* isolates examined in this study for their hydrocarbon degradation capacities can be used as an effective tool to deal with hydrocarbon impurities. Psychrotolerance was previously observed in *Bacillus* sp., isolated from oil-contaminated soils in the study performed by Zhan et al [30]. A total of 597 *Bacillus* isolates found in “Bioengineering Culture Collection” of Ege University, Izmir, Turkey were inoculated on CASO agar plates and incubated at 10 and 7 °C temperature for 21 days. These temperature ranges were determined by literature review [14],[19],[31]. The results determined that out of 597 isolates 388 (65%) isolates have shown growth at 10 °C and 134 (22%) isolates have shown the capacities to grow at 7 °C. These 134 isolates were tested for their growth capabilities at 4 °C and only 25 isolates have shown growth at 4 °C. The upper temperature limits of 134 psychrotolerant isolates were determined by incubating them at 43 and 47 °C. Out of 134 psychrotolerant isolates 132 were capable of growing at 43 °C and 99 isolates have shown growth at 47 °C. In a previous study, temperature limits for *Bacillus cereus*, *Bacillus mycoides*, *Bacillus thuringiensis* and *Bacillus anthracis* were determined and the isolates grown at 7 °C or below were considered as psychrotolerant [14]. Another study on *Bacillus* sp. recovered from the tropical Atlantic and Antarctic and Arctic oceans was conducted at various temperatures (1, 2, 4, 12, 18, 24, 30 and 37 °C). The results determined that most of the isolates have an upper limit of 30 °C and a lower limit of 8 °C, dissimilar to our study [32].

Hydrocarbon degradation capabilities of psychrotolerant isolates were initially determined on Bushnell Haas agar. Results depicted that out of 134 psychrotolerant isolates only 18 (13%) isolates have shown an absence of growth in the presence of hydrocarbons and 116 (87%) isolates have shown hydrocarbon degradation abilities against at least one of the selected hydrocarbon. A total of 47 isolates have shown the capabilities to degrade all three selected hydrocarbons (data not shown here). These 47 isolates were further tested for hydrocarbon degradation abilities in broth medium under static culture conditions and the results were represented as biomass (cfu ml^−1^) (Table 1). In literature biodegradation studies were mostly carried out by shaking culture methods typically at 150–200 rpm [33],[34],[35]. However, static culture conditions are more appropriate to reflect naturally existing ecosystem. According to the results obtained from static culture experiments five best isolates have been selected and grown in the presence of 1% crude oil as a carbon source in Bushnell Haas broth media under shaking culture conditions. Keeping in mind the temperature tolerance of psychrotolerant microbes and considering the weather conditions of natural environments, incubation temperature was set to 20 °C in our study. Biomass obtained after 5 and 10 days in shaking culture experiments are presented in Table 2. Trejo-Hernandez et al [33] determined the biomass after five days as 4.0 × 10^5^ cfu ml^−1^ in shaking culture in the presence of crude oil as carbon source. On 15^th^ day this number reached to 10^9^ cfu ml^−1^. In contrast to previous reports, our isolate *Bacillus* Ege B.6.2i has shown higher growth rate at the end of 5^th^ day (1.2 × 10^7^ cfu ml^−1^), the rate gradually increased and reached to 7.4 × 10^8^ cfu ml^−1^ at 10^th^ day. Similarly *Bacillus* Ege B 1.4 and *Bacillus* Ege B 39.1 isolates have also shown higher biomass production at 5^th^ days as compared to literature (Table 2).

**Table 1.** Degradation of hydrocarbons by psychrotolerant *Bacillus* isolates in static culture.

**Isolate code**	**Biomass (cfu ml^−1^) of bacteria calculated after 21 days**		
	**Hexadecane**	**Naphthalene**	**Mineral oil**
*Bacillus* Ege B.12.3i	5.5 × 10^4^	4.0 × 10^4^	1.9 × 10^4^
*Bacillus* Ege B.42.5	5.6 × 10^6^	1.4 × 10^7^	–
*Bacillus* Ege B.13.3i	–	–	2.9 × 10^5^
*Bacillus* Ege B.27.1	7.6 × 10^4^	–	1.8 × 10^4^
*Bacillus* Ege B.27.2	1.2 × 10^6^	1.1 × 10^7^	2.4 × 10^6^
*Bacillus* Ege B.25.3	4.1 × 10^5^	1.5 × 10^6^	–
*Bacillus* Ege B.25.4	7.5 × 10^4^	1.7 × 10^5^	1.8 × 10^4^
*Bacillus* Ege B.9.2i	6.3 × 10^5^	–	1.7 × 10^6^
*Bacillus* Ege B.6.3i	1.1 × 10^6^	1.0 × 10^6^	1.3 × 10^5^
*Bacillus* Ege B.45.1	5.1 × 10^6^	5.3 × 10^6^	9.3 × 10^6^
*Bacillus* Ege B.22.2	1.6 × 10^7^	7.0 × 10^7^	–
*Bacillus* Ege B 44.4	5.3 × 10^5^	4.8 × 10^5^	3.5 × 10^5^
*Bacillus* Ege B 1.26	3.7 × 10^6^	2.2 × 10^6^	1.2 × 10^5^
*Bacillus* Ege B 1.2k	1.4 × 10^6^	1.7 × 10^6^	2.3 × 10^6^
*Bacillus* Ege B 1.3F	–	2.0 × 10^4^	–
*Bacillus* Ege B 1.2F	7.6 × 10^5^	2.0 × 10^5^	1.3 × 10^5^
*Bacillus* Ege B 2.8.2	1.5 × 10^6^	1.9 × 10^6^	1.3 × 10^5^
*Bacillus* Ege B 36.3	3.0 × 10^5^	5.5 × 10^5^	1.6 × 10^5^
*Bacillus* Ege B 31.4	4.7 × 10^5^	8.3 × 10^5^	1.0 × 10^5^
*Bacillus* Ege B 39.1	2.2 × 10^7^	2.0 × 10^7^	1.1 × 10^7^
*Bacillus* Ege B 29.1	1.7 × 10^6^	2.0 × 10^6^	1.8 × 10^6^
*Bacillus* Ege B 23.2i	1.5 × 10^6^	1.6 × 10^6^	1.3 × 10^6^
*Bacillus* Ege B 1.25k	1.4 × 10^6^	1.7 × 10^6^	1.1 × 10^6^
*Bacillus* Ege Bi 1.2	1.8 × 10^6^	2.6 × 10^5^	3.0 × 10^5^
*Bacillus* Ege Bi 1.3	3.2 × 10^6^	1.3 × 10^6^	5.9 × 10^6^
*Bacillus* Ege Bi 1.9	3.8 × 10^6^	4.5 × 10^6^	4.0 × 10^6^
*Bacillus* Ege B 2d.7	2.8 × 10^6^	2.5 × 10^6^	–
*Bacillus* Ege B 6.2i	1.1 × 10^7^	4.0 × 10^6^	8.8 × 10^6^
*Bacillus* Ege B 1.3	–	–	–
*Bacillus* Ege B.1.4Ka	3.7 × 10^6^	3.7 × 10^6^	2.7 × 10^6^
*Bacillus* Ege B 12.3i	–	–	–
*Bacillus* Ege B 25.5	–	–	–
*Bacillus* Ege B 2d.9	–	–	–
*Bacillus* Ege B 8.4.F	–	1.0 × 10^6^	–
*Bacillus* Ege B.8.1.F	1.5 × 10^5^	–	5.2 × 10^5^
*Bacillus* Ege B.17.2i	2.3 × 10^5^	2.6 × 10^5^	–
*Bacillus* Ege B.24.4	–	2.2 × 10^5^	–
*Bacillus* Ege B.24.5	2.8 × 10^6^	8.4 × 10^5^	–
*Bacillus* Ege B.11.8i	2.4 × 10^6^	3.7 × 10^6^	7.0 × 10^5^
*Bacillus* Ege B.1.18k	1.7 × 10^5^	4.2 × 10^5^	–
*Bacillus* Ege B 31.2	2.3 × 10^4^	6.0 × 10^5^	–
*Bacillus* Ege B 42.2	1.5 × 10^5^	1.5 × 10^5^	1.5 × 10^4^
*Bacillus* Ege B 19.1i	2.5 × 10^4^	1.5 × 10^5^	3.3 × 10^4^
*Bacillus* Ege B 43.4	2.0 × 10^4^	8.0 × 10^5^	–
*Bacillus* Ege B 22.3i	4.1 × 10^5^	4.2 × 10^6^	5.0 × 10^4^
*Bacillus* Ege B 44.3	7.5 × 10^5^	6.8 × 10^5^	6.7 × 10^4^
*Bacillus* Ege B 40.5	3.5 × 10^4^	2.0 × 10^5^	–

(–, <10^4^ cfu ml^−1^)

**Table 2.** Crude oil degradation by psychrotolerant *Bacillus* isolates in shaking culture.

**Isolate**	**Biomass in presence of crude oil**	
	**5^th^ day**	**10^th^ day**
*Bacillus* Ege B.45.1	5.1 × 10^5^	8.3 × 10^5^
*Bacillus* Ege B.39.1	5.7 × 10^6^	1.3 × 10^7^
*Bacillus* Ege Bi.1.9	5.6 × 10^5^	2.2 × 10^6^
*Bacillus* Ege B.6.2i	1.2 × 10^7^	7.4 × 10^8^
*Bacillus* Ege B.1.4Ka	2.6 × 10^6^	9.6 × 10^6^

The degradation activity of the bacteria changes according to the structure of the substrate provided [36]. Depending on the differences in structure a priority pattern has been assessed for the biodegradation of hydrocarbons. This pattern can be represented as n-alkanes > branched alkanes > low molecular weight aromatics > cycloalkanes [29]. Therefore, while comparing crude oil to hexadecane, naphthalene and mineral oil as a substrate in bacterial growth medium, crude oil was found to be the most beneficial carbon source. The complex structure of crude oil consists of various compounds which provide bacteria a chance to degrade if not all then at least a few of these compounds. With this reason in mind, isolates were grown in the presence of crude oil in shaking culture. According to the results isolate *Bacillus* Ege B.8.4.F has shown no growth in the presence of hexadecane and mineral oil while in the presence of crude oil, the biomass reached to 1.2 × 10^6^ cfu ml^−1^ in a short period of just 5 days (Table 2). *Bacillus* Ege B.8.1.F has shown 1.5 × 10^5^ cfu ml^−1^ after 21 days in the presence of hexadecane as carbon source while in the presence of crude oil the count was noted as 9.8 × 10^5^ cfu ml^−1^ after 5 days. These results depicted that the growth characteristics vary according to the carbon sources as previously mentioned in literature [36].

**Figure 1.**
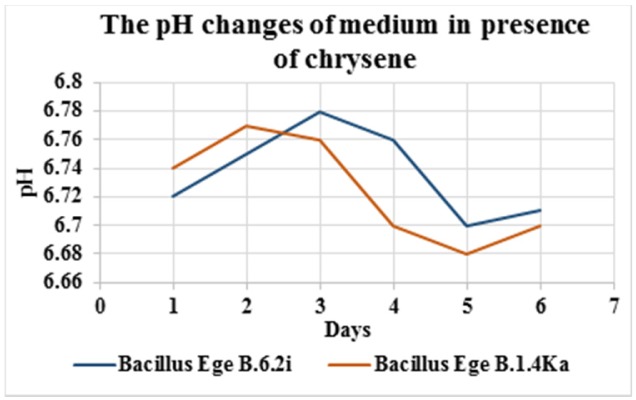
Graph showing pH changes of chrysene containing medium for *Bacillus* Ege B.6.2i and *Bacillus* Ege B.1.4Ka.

**Figure 2.**
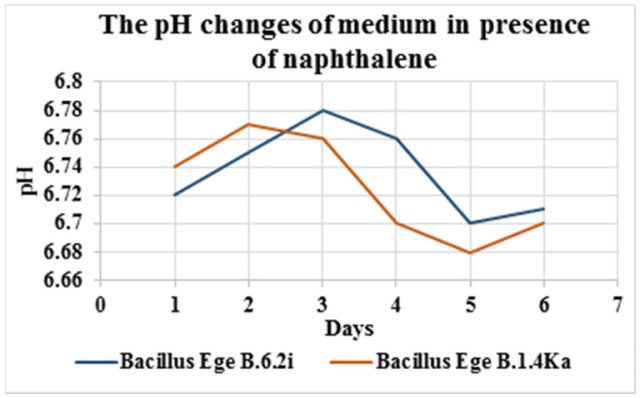
Graph showing pH changes of naphthalene containing medium for *Bacillus* Ege B.6.2i and *Bacillus* Ege B.1.4Ka.

After the screening of biodegradation abilities in agar and broth mediums (static and shaker culture) on the basis of results *Bacillus* Ege B.6.2i and *Bacillus* Ege B.1.4.Ka were selected as two best biodegrading isolates. These two isolates were tested for their biodegradation potential against PAHs (naphthalene and chrysene); more specific and toxic group of hydrocarbons. Isolates were incubated for 6 days at 20 °C in shaking culture. The pH changes of the broth (Figure 1 and Figure 2), dry mass (Table 3) and viable cell counts (cfu ml^−1^) (Table 4) were calculated on daily bases. In a previous study pH changes were observed in the Bushnell Hass medium for 15 days and a minor change in pH has been observed in contrast to our study [21]. Dry mass for both isolates was gradually increased until 6 days and *Bacillus* Ege B.1.4Ka has shown the maximum dry mass (0.51 g 50 ml^−1^). In a study conducted with *Bacillus* and *Pseudomonas* sp. using chrysene as a carbon source dry mass was noted as 0.2 g 50 ml^−1^ for *Bacillus* sp. and 0.3 g 50 ml^−1^ for *Pseudomonas* sp. after 7 days, which is lesser as compared to our results [35].

**Table 3.** Dry mass of *Bacillus* Ege B.6.2i and *Bacillus* Ege B.1.4Ka in presence of PAHs.

		**Dry mass measures g 50 ml^−1^**					

**Hydrocarbon**	**Isolate**	**Day 1**	**Day 2**	**Day 3**	**Day 4**	**Day 5**	**Day 6**
Naphthalene	*Bacillus* Ege B.6.2i	0.02	0.04	0.21	0.35	0.33	0.30
	*Bacillus* Ege B.1.4Ka	0.01	0.03	0.32	0.38	0.43	0.51
Chrysene	*Bacillus* Ege B.6.2i	0.01	0.05	0.20	0.20	0.30	0.40
	*Bacillus* Ege B.1.4Ka	0.01	0.02	0.30	0.40	0.40	0.30

**Table 4.** Viable count of *Bacillus* Ege B.6.2i and *Bacillus* Ege B.1.4Ka in presence of PAHs.

**Days**	**cfu ml^−1^ in presence of naphthalene**		**cfu ml^−1^ in presence of chrysene**	
	*Bacillus* Ege B.1.4Ka	*Bacillus* Ege B.6.2i	*Bacillus* Ege B.1.4Ka	*Bacillus* Ege B.6.2i
1	1.1 × 10^5^	1.5 × 10^5^	3.8 × 10^5^	4.0 × 10^4^
2	1.0 × 10^5^	3.4 × 10^5^	4.1 × 10^5^	8.5 × 10^4^
3	1.8 × 10^5^	7.1 × 10^5^	6.4 × 10^5^	3.3 × 10^5^
4	4.5 × 10^5^	3.0 × 10^5^	5.8 × 10^5^	3.1 × 10^5^
5	5.5 × 10^5^	1.8 × 10^5^	7.4 × 10^5^	3.1 × 10^5^
6	8.6 × 10^5^	1.2 × 10^5^	1.7 × 10^5^	1.2 × 10^6^

**Figure 3.**
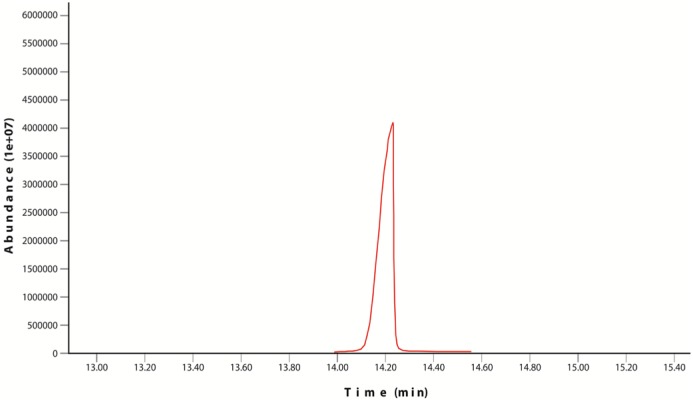
GC-MS analysis of chrysene for *Bacillus* Ege B.1.4Ka.

**Figure 4.**
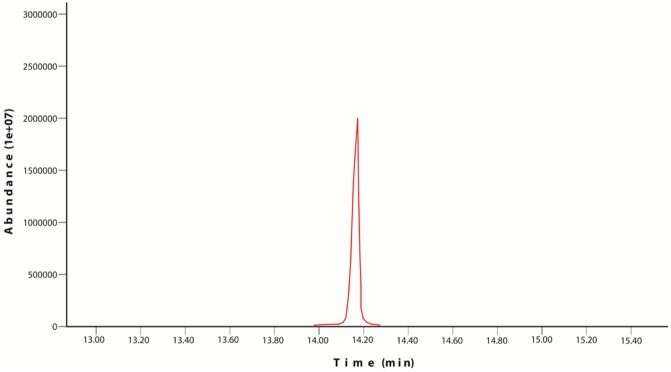
GC-MS analysis of chrysene for *Bacillus* Ege B.6.2i.

**Figure 5.**
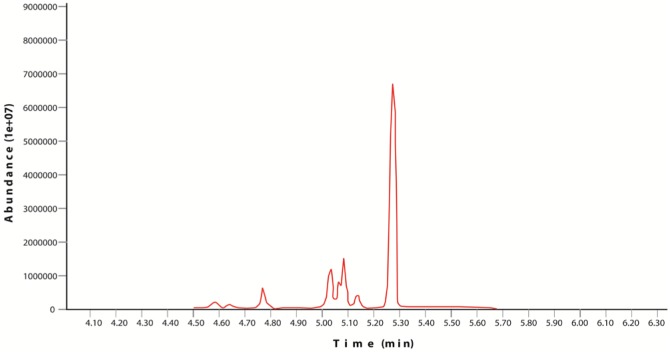
GC-MS analysis of naphthalene for *Bacillus* Ege B.1.4Ka.

**Figure 6.**
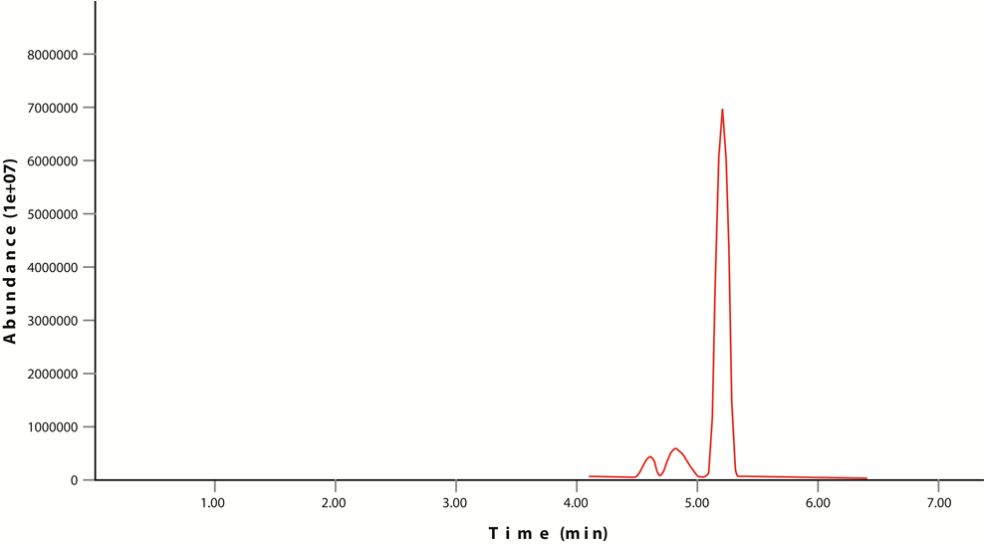
GC-MS analysis of naphthalene for *Bacillus* Ege B.6.2i.

Chrysene and naphthalene residual amounts were measured by GC-MS and results were mentioned in the Table 5 and Table 6. In control flask 80% recovery has been observed for chrysene while for naphthalene this value is recorded up to 86%. According to the results of samples, isolate *Bacillus* Ege B.6.2i has shown better results in terms of removal of chrysene from the medium than isolate *Bacillus* Ege B.1.4Ka. GC-MS results were represented in graphs (Figure 3 and Figure 4). GC-MS analysis of naphthalene for *Bacillus* Ege B.1.4Ka and *Bacillus* Ege B.6.2i were shown in Figure 5 and Figure 6. In past studies PAH analysis has been carried out using GC-MS, with similar kind of methods [2],[21],[22],[33],[34],[35]. According to the data in the literature a recovery rate of 88% ± 2 was reported in control flasks using chrysene as substrate [35]. In another study chrysene degradation capacity of *Bacillus* and *Pseudomonas* sp. has been studied by GC analysis and degradation efficacy was recorded as 17% for *Bacillus* sp. and 15% for *Pseudomonas* sp. [35]. Christova et al [22] worked with n-hexadecane and naphthalene degrading *Bacillus* *subtilis* 22BN strain and studied the relation between hydrocarbon degradation and bio-surfactant (rhamnolipid) production.

**Table 5.** GC-MS analysis of chrysene degradation capacity of *Bacillus* Ege B.6.2i and *Bacillus* Ege B.1.4Ka.

**Isolate**	**Initial chrysene quantity (mg L^−1^)**	**Chrysene quantity recovered from GC-MS (mg L^−1^)**	**Recovery (%)**	**Yield (%)**
Control	50	40	80	–
*Bacillus* Ege B.6.2i	50	15.45	–	60
*Bacillus* Ege B.1.4Ka	50	25.5	–	36

**Table 6.** GC-MS analysis of naphthalene degradation capacity of *Bacillus* Ege B.6.2i and *Bacillus* Ege B.1.4Ka.

**Isolate**	**Initial naphthalene quantity (mg L^−1^)**	**Naphthalene quantity recovered from GC-MS (mg L^−1^)**	**Recovery (%)**	**Yield (%)**
Control	100	86	86	–
*Bacillus* Ege B.6.2i	100	59.05	–	33
*Bacillus* Ege B.1.4Ka	100	38.9	–	55

In most of the previous studies emulsification activity measurement is done by using the method of Cooper and Goldenberg (1987) [23], but few researchers have modified this method in their works. These modifications include the use of different hydrophobic substrate like toluene, xylene, mineral oil or crude oil as an alternative of kerosene [2],[18],[35],[37]. In our study Cooper and Goldenberg's method [23] is modified and the transfer tube volume is reduced by half (3/2 ml) while mineral oil was used as hydrophobic substrate instead of kerosene. Emulsification indexes calculated in past studies are in accordance with our results [35]. Emulsification index values were found to be higher when high viable counts were present in the chrysene biodegradation experiment (Table 7). Literature shows high emulsification activities up to 53% in presence of chrysene for bio-surfactant production. *Bacillus* Ege B.1.4Ka isolates showed naphthalene removal of 55% and the emulsifying activity was measured as 40%. Naphthalene degradation activity was found higher for *Bacillus* Ege B.1.4Ka while, emulsification activity was higher for isolates *Bacillus* Ege B.6.2i (Table 7). In case of high emulsification activity the increase in biodegradation activity was observed in our results. All these assessments are in line with the literature [18],[35].

**Table 7.** Viable count, GC-MS analysis and emulsification activities in presence of PAHs.

**PAH**	**Isolate**	**Viable cell count (cfu ml^−1^)^a^**	**Hydrocarbon removal (%)**	**Emulsification activity (%)^b^**
**Naphthalene**	*Bacillus* Ege B.6.2i	1.2 × 10^5^	33	37
	*Bacillus* Ege B.1.4Ka	8.6 × 10^5^	55	40
**Chrysene**	*Bacillus* Ege B.6.2i	1.2 × 10^6^	60	50
	*Bacillus* Ege B.1.4Ka	1.7 × 10^5^	36	33

^a^, after 6 days; ^b^, after 2 days.

Naphthalene was the only hydrocarbon degraded by isolates under static as well as shaking culture. In static and shaking culture experiment results depicted that shaking culture experiment has shown better results in less time. In a previous study with static and shaking culture conditions naphthalene degradation yield was recorded as 50% after 24 hours in static culture, on the other hand, for shaking culture this value was reported as 95% [38]. However, determining best culture condition for highest biodegradation was not the goal of our present study, but the purpose was to find potential bioremediation agents capable of degrading hydrocarbons in natural environment under unfavourable conditions. Therefore, static culture results are more important as the static culture conditions more closely reflect the natural environmental condition. Accumulation of hydrophobic contaminants on the surface of the medium in a static culture environment would be a more realistic scenario [39]. In case of accidents and leaks occurring in the sea, resulting in spilled oil, it is not possible to provide ideal ecosystem to accelerate the disintegration of leaked oil therefore, isolates showing good results under static culture are believed to be more effective to remove the contaminants from ecosystem under unfavourable conditions. In our study hydrocarbon concentrations are kept very high in the growth medium to create an environment close to accidental oil spill. Studies are found where up to milligram concentration of hydrocarbon had been used in the growth medium [22].

*Bacillus* isolates are being considered as an effective organism for biodegradation of hydrocarbons. A survey conducted in Lagos region of Nigeria, nine different bacteria isolated from hydrocarbon contaminated tropical river were identified as hydrocarbon degraders including *Pseudomonas fluorescens*, *P. aeruginosa*, *Bacillus subtilis*, *Bacillus sp*., *Alcaligenes* sp., *Acinetobacter lwoffi*, *Flavobacterium* sp., *Micrococcus roseus* and *Corynebacterium* sp. [40]. In another study *B.subtilis* DM-04, *P.aeruginosa* M and NM isolates were examined for crude oil biodegradation and all isolates have been recommended as effective bioremediation agents [41]. With other microorganisms different *Bacillus* species have also been recognized as effective biodegrading agents in many past studies [2],[36]. In addition to other studies our work also recommends effectiveness of *Bacillus* sp. for biodegradation but in contrast to other finding instead of using mesophilic isolates this study has been conducted with psychrotolerant isolates. Similar to our findings, in a study Antarctic marine bacteria have been isolated and biodegrading studies have been conducted at 4 and 15 °C [20]. Margesin and Schinner [42] have studied diesel oil biodegradation abilities of RM8/11 psychrotolerant isolate at 10 °C with different concentrations of bio-surfactants. In another study hydrocarbon degradation psychrotolerant bacteria isolated from the surface of Antarctic sea were studied at 4 °C for their diesel oil degradation properties [13]. In recent studies, it was reported that a number of aerobic bacteria have been used to biodegrade naphthalene with several pathways and metabolic diversities [7]. For example, Zhuang et al [43] found that *Bacillus naphthovorans* is capable to degrade the anthracene from the oil-contaminated tropical marine sediments with a yield of 77% at 43 °C. Lin et al [7] found 78% anthracene removal by biodegradation process using *Bacillus fusiformis* at 27–30 °C. Swaathy et al [44] observed 67% anthracene removal using *Bacillus licheniformis* MTCC 5514 at 28 °C. Dhote et al [35] investigated the chrysene biodegradation with a yield of 72% at 28 °C under aerobic conditions with enzymes namely catechol 1,2-dioxygenase and catechol 2,3-dioxygenase catalyzing the chrysene mineralization. Ukiwe et al [45] obtained 45% chrysene removal with different bacteria isolates. Cold-adapted indigenous microorganisms play a significant role in the in-situ biodegradation of hydrocarbons in cold environments, where temperatures often coincide with their growth temperature range. The capability to utilize a wide range of hydrocarbons is advantageous for the treatment of mixed pollutions.

## 4. Conclusions

The strains investigated in this study could grow and degrade hydrocarbons over a broad temperature range. The application of such degraders is advantageous in cold and temperate environments that undergo (diurnal and/or seasonal) thermal ﬂuctuations. Such strains are useful at low temperature and consequently can be used for low-energy treatment of industrial efﬂuents contaminated with hydrocarbons. In addition, they could also be useful for the construction of biosensors for the selective, sensitive and rapid monitoring or in situ analysis of pollution. In brief, psychrotolerant *Bacillus* isolates have shown a great potential for the low-temperature biodegradation of hydrocarbons. Considering their great metabolic versatility, the contribution of these bacteria to the biodegradation of hydrocarbons in the environment and as a potentially exploitable enzyme producer is much more important than currently expected.
